# Evaluation of left ventricular myocardial stratified strain in patients with Kawasaki disease using two-dimensional speckle tracking imaging

**DOI:** 10.3389/fcvm.2022.899945

**Published:** 2022-07-27

**Authors:** Jinling Hu, Qiaojin Zheng, Weidong Ren

**Affiliations:** Department of Ultrasound, Shengjing Hospital of China Medical University, Shenyang, China

**Keywords:** Kawasaki disease, echocardiography, speckle tracking imaging, left ventricular function, stratified strain

## Abstract

Kawasaki disease (KD) is an acute autoimmune self-limited disease of unknown etiology. We aimed to evaluate the left ventricular myocardial stratified strains in children with KD in different stages using two-dimensional speckle tracking imaging, and to find the index that can early predict myocardial function injury in children with KD. A total of 73 children with KD were divided into acute, convalescent, and chronic stages according to the disease course. All children had no coronary artery damage. Further, 64 normal children were selected as the control group. The longitudinal and circumferential strain peaks of each myocardium were recorded, and the left ventricular global longitudinal strain (LVGLS), global circumferential strain (LVGCS), and transmural gradient between endocardium and epicardium (ΔGLS, ΔGCS) were calculated. Compared with the control group, LVGLS, GLS-Endo, GLS-Mid, GLS-Epi, and ΔGLS decreased in acute KD; LVGLS, GLS-Endo, GLS-Mid, GLS-Epi, ΔGLS, LVGCS, GCS-Mid, and GCS-Epi decreased in the convalescent stage; and only GLS-Endo was lower in children with chronic KD (*P* < 0.05). ROC curve was used to calculate the stratified strain parameters so as to predict left ventricular systolic function in children with acute KD; the area under the LVGLS curve was the largest (AUC = 0.953, *P* < 0.001). When the cutoff value of LVGLS was −19.89%, the sensitivity and specificity were 95.8% and 83.2%, respectively.

**Conclusions:**

The systolic function of the whole layer of the myocardium decreased to varying degrees in children with KD. With the extension of the disease course, the myocardial function gradually recovered, but the subendocardial myocardium was still damaged. LVGLS could identify the abnormity of left ventricular contractility in patients with KD at the acute stage. Thus, it has the promising prospect of clinical diagnosis.

## Introduction

Kawasaki disease (KD), also known as mucocutaneous lymph node syndrome (MCLS), is an acute febrile disease with systemic small- and medium-vessel vasculitis (1). It is an acute autoimmune self-limited disease of unknown etiology, mainly affecting children aged <5 years (1). The most serious complication in children with KD is coronary artery lesions (CAL), which leads to myocardial ischemia and cardiac dysfunction; it is one of the main causes of acquired heart disease in children (2). For children with atypical KD, delayed diagnosis significantly increases the risk of CAL. Although CAL is the main cause of late death in children with KD, children with KD can also be complicated with pericardial effusion, cardiac valvular insufficiency, ventricular dilatation, reduced systolic function, arrhythmia, and other complications of non-coronary artery injury (3). Myocardial tissue biopsy in children with KD has confirmed that the myocardial cells in the acute stage show edema and myocardial inflammatory damage (4, 5). For KD children without coronary artery injury, myocardial inflammatory injury is an important factor for left ventricular systolic dysfunction; also, diffuse myocarditis is an important cause of myocardial fibrosis and left ventricular systolic dysfunction in children with KD (6). The latest international guidelines on KD suggest that evaluating left ventricular systolic function in children with KD is an essential auxiliary indicator of the disease (7). The incidence of KD in children continues to increase and the age at onset is reducing every year, making the diagnosis difficult. If not diagnosed and treated in time, the prognosis of children can be poor. Therefore, early diagnosis and timely treatment have a crucial impact on the prognosis of children. Nowadays, routine echocardiography is the most commonly used method to evaluate left ventricular systolic dysfunction clinically. However, the decrease of ejection fraction evaluated by routine echocardiography often indicates the clinical status of the disease. Therefore, the detection of subclinical systolic dysfunction in children with KD is limited.

Two-dimensional speckle tracking imaging (2D-STI) is a newly developed method to evaluate the local and global systolic and diastolic functions of the myocardium in clinical ultrasound examination (8, 9). Recent studies have confirmed that 2D-STI is not affected by acoustic beam and has no angle dependence. It can accurately and sensitively evaluate the overall and local myocardial function of adults and children (10), and hence, is better than routine echocardiography. Few studies have been conducted on left ventricular myocardial function in children with KD in different stages. In this study, 2D-STI was used to examine the changes in the three-layer myocardium of the left ventricle in children with KD in different stages. The study aimed to evaluate the accuracy and clinical value of stratified strain in assessing the abnormal early myocardial systolic function of children with KD, so as to provide the basis for clinical prevention and timely treatment.

## Materials and methods

### Patients

A total of 73 children (37 male and 36 female) with KD, aged 14 months to 10 years (average age 4.24 ± 2.17 years), were admitted to the Department of Pediatric Cardiology at Shengjing Hospital of China Medical University from January 2021 to December 2021. All children diagnosed on the basis of the clinical criteria for KD (11), and all of them had no coronary artery damage (Z-score < 2.0). The exclusion criteria were as follows: (1) Children with hematological diseases, (2) children with congenital heart disease, (3) children with severe liver and kidney dysfunction, (4) children with cardiopulmonary diseases and genetic metabolic diseases, and (5) children not cooperating with the examination. According to the disease course, 73 children with KD were divided into 24 cases in the acute stage (within 2 weeks of onset, no drug intervention), 26 cases in the convalescent stage (1–3 months after onset, drug intervention), and 23 cases in the chronic stage (disease course > 3 months, drug intervention). Further, 64 normal children (31 male and 33 female), aged 2 months to 10 years (average age 5.01 ± 2.82 years) who underwent physical examination in the hospital during the same period were selected as the control group. Whole blood, plasma and serum samples were collected from all patients with KD before any treatment with intravenous immunoglobulin in acute stage. These parameters included the following: white blood cell count (WBC), C-reactive protein (CRP), erythrocyte sedimentation rate (ESR), aspartate aminotransferase (AST), and alanine aminotransferase (ALT). This study was approved and reviewed by the Ethics Committee of China Medical University, and all methods were performed in accordance with the principles outlined in the Declaration of Helsinki. Informed consent was obtained from all subjects or, if subjects are under 16, from a parent and/or legal guardian.

### Instruments and methods

A Philips EPIQ7c (Andover, MA, USA) color Doppler ultrasound machine was used; an S8-3 or S5-1 probe was selected for the ultrasonic examination according to the individual size of the participant. When the patient was quiet, the left or supine position was taken, and the three-lead ECG was connected to the chest. Before data collection, the gain, depth, sampling frame, and focus position were adjusted to make the image as clear as possible. Left ventricular end-diastolic volume (LVEDV), left ventricular end-systolic volume (LVESV), and left ventricular ejection fraction (LVEF) were obtained using the two-plane Simpson method. The left ventricular posterior wall (LVPW) and the interventricular septum (IVS) were measured on the standard parasternal left ventricular long-axis section, and the heart rate was measured in real-time. At least three to five dynamic cardiac cycle images were collected from the basal segment of the left ventricular short axis (mitral valve level), the middle segment of the left ventricular short axis (papillary muscle level), the apical segment of the left ventricular short axis, apical four-chamber view, apical two-chamber view, and apical three-chamber view, and stored in DICOM (Digital Imaging and Communication in Medicine) format.

### STI analysis

The stored dynamic images of the six sections were successively retrieved and processed using the instrument software (Qlab version 13.0, Speckle Tracking; Philips) to select the cardiac cycle in which both the endocardium and epicardium were clearly and effectively displayed. The endocardium was mapped using aCMQ speck tracking software, which automatically traced the myocardium The longitudinal and circumferential strain curves of the subendocardial myocardium, middle myocardium, and subepicardial myocardium corresponding to each section were obtained systematically. The global longitudinal strain in subendocardial myocardium (GLS-Endo), global longitudinal in middle myocardium (GLS-Mid), global longitudinal in subepicardial myocardium (GLS-Epi), global circumferential strain in subendocardial myocardium (GCS-Endo), global circumferential in middle myocardium (GCS-Mid), and global circumferential in subepicardial myocardium (GCS-Epi) were recorded, and the left ventricular global longitudinal strain (LVGLS) and left ventricular global circumferential strain (LVGCS) were calculated. The difference between the corresponding outer and intimal strains was calculated to obtain the transmural strain gradient, that is, the longitudinal transmural strain gradient (ΔGLS) and the circumferential transmural strain gradient (ΔGCS) of the left ventricular myocardium. All stratified strain parameters were averaged from the analysis results of three different cardiac cycles, and all images were analyzed by two ultrasound physicians (JH and QZ).

### Statistical analysis

All statistical analyses were performed using SPSS software (Statistical Product and Service Solutions Company, Chicago) version 23.0. The general clinical data, routine echocardiographic parameters, and left ventricular stratified and global strains were statistically analyzed following normal distribution, which was described by the mean ± standard deviation ($\bar{x}$ ± s). One-way analysis of variance was used to compare the general clinical data, routine echocardiographic parameters, and left ventricular stratified strain and global strain parameters between children with KD during different periods and control children. The LSD (Least Significant Difference) –*t*-test was used for further pairwise comparison. Receiver operating characteristic (ROC) curves were applied to calculate the area under the curve (AUC) of stratified strain parameters to predict left ventricular systolic function in children with acute KD. The values were considered significantly different at *P* < 0.05.

## Results

### Comparison of general clinical data

In the acute stage, echocardiography showed pericardial effusion in one patient, mitral regurgitation in five patients, tricuspid regurgitation in six patients, arrhythmia in two patients. In the control group, mitral regurgitation in two patients, tricuspid regurgitation in three patients, arrhythmia in two patients. No significant differences were found in age, height, and AST between the KD and control groups (Table 1).

**Table 1:** Comparison of general clinical data between the two groups.

**Group**	**Cases**	**Age** **(years)**	**Height** **(cm)**	**Weight** **(Kg)**	**body surface area** **(m**^2^**)**	
KD group	73					
Acute stage	24	3.63 ± 2.21^a^	102.08 ± 19.40^b^	16.17 ± 5.44^c^	0.48 ± 0.25^d^	
Convalescent stage	26	4.58 ± 1.90	114.58 ± 15.96	21.62 ± 9.78	0.70 ± 0.33	
Chronic stage	23	4.50 ± 2.37	111.13 ± 18.44	25.17 ± 12.69	0.82 ± 0.55	
Control group	64	5.01 ± 2.82	113.22 ± 22.88	20.98 ± 9.21	0.71 ± 0.43	
*F*		1.810	2.039	3.598	2.819	
*P*		0.148	0.112	0.015*	0.042*	
**Group**	**Cases**	**WBC** **(×10^9^/L)**	**CRP** **(mg/L)**	**ESR** **(mm/h)**	**AST** **(U/L)**	**ALT** **(U/L)**
KD group	73					
Acute stage	24	13.54 ± 6.90^c^	82.33 ± 54.58^d^	62.21 ± 28.71^c^	31.21 ± 21.96^a^	39.71 ± 55.97^a^
Convalescent stage	26	10.56 ± 5.76	72.16 ± 59.51^d^	51.23 ± 29.27^d^	26.81 ± 17.63	34.73 ± 34.09^a^
Chronic stage	23	9.83 ± 3.76	26.71 ± 19.94^a^	24.23 ± 17.72^a^	27.22 ± 9.56	23.96 ± 6.10
Control group	64	8.56 ± 2.85	4.09 ± 2.36	9.70 ± 5.79	23.97 ± 8.18	20.34 ± 10.99
*F*		7.151	40.653	57.132	1.672	3.431
*P*		<0.001*	<0.001*	<0.001*	0.176	0.019*

*P < 0.05. a, Compared with the control group, the difference was statistically significant (P < 0.05); b, Compared with the control group and children with KD in convalescent stage, the differences were statistically significant (P < 0.05); c, Compared with the control group, children with KD in convalescent stage and chronic stage, the differences were statistically significant (P < 0.05); d, Compared with the control group and children with KD in chronic stage, the differences were statistically significant (P < 0.05).

Comparison of routine echocardiographic parameters: No statistically significant differences were observed in routine echocardiographic parameters LVEDV, LVESV, LVEF, IVS, and LVPW between the two groups (Table 2).

**Table 2:** Comparison of routine echocardiographic parameters between the two groups ($\bar{x}$ ± s).

**Group**	**Cases**	**LVEDV(mL)**	**LVESV(mL)**	**LVEF(%)**	**IVS(mm)**	**LVPW(mm)**
KD group	73					
Acute stage	24	38.30 ± 12.50*	12.65 ± 4.64	67.17 ± 3.44	4.28 ± 0.80	4.23 ± 0.80
Convalescent stage	26	46.69 ± 14.72	15.42 ± 5.16	66.73 ± 4.49	4.36 ± 0.78	4.40 ± 0.78
Chronic stage	23	47.17 ± 13.94	15.30 ± 5.36	67.61 ± 4.34	4.61 ± 0.86	4.56 ± 0.77
Control group	64	44.14 ± 16.67	14.52 ± 5.89	66.88 ± 3.79	4.52 ± 0.84	4.48 ± 0.86
*F*		1.727	1.333	0.254	0.885	0.786
*P*		0.165	0.266	0.858	0.451	0.504

*Compared with the children with KD in convalescent stage, the differences was statistically significant (P < 0.05).

### Comparison of 2D-STI parameters

**Figure 1:**
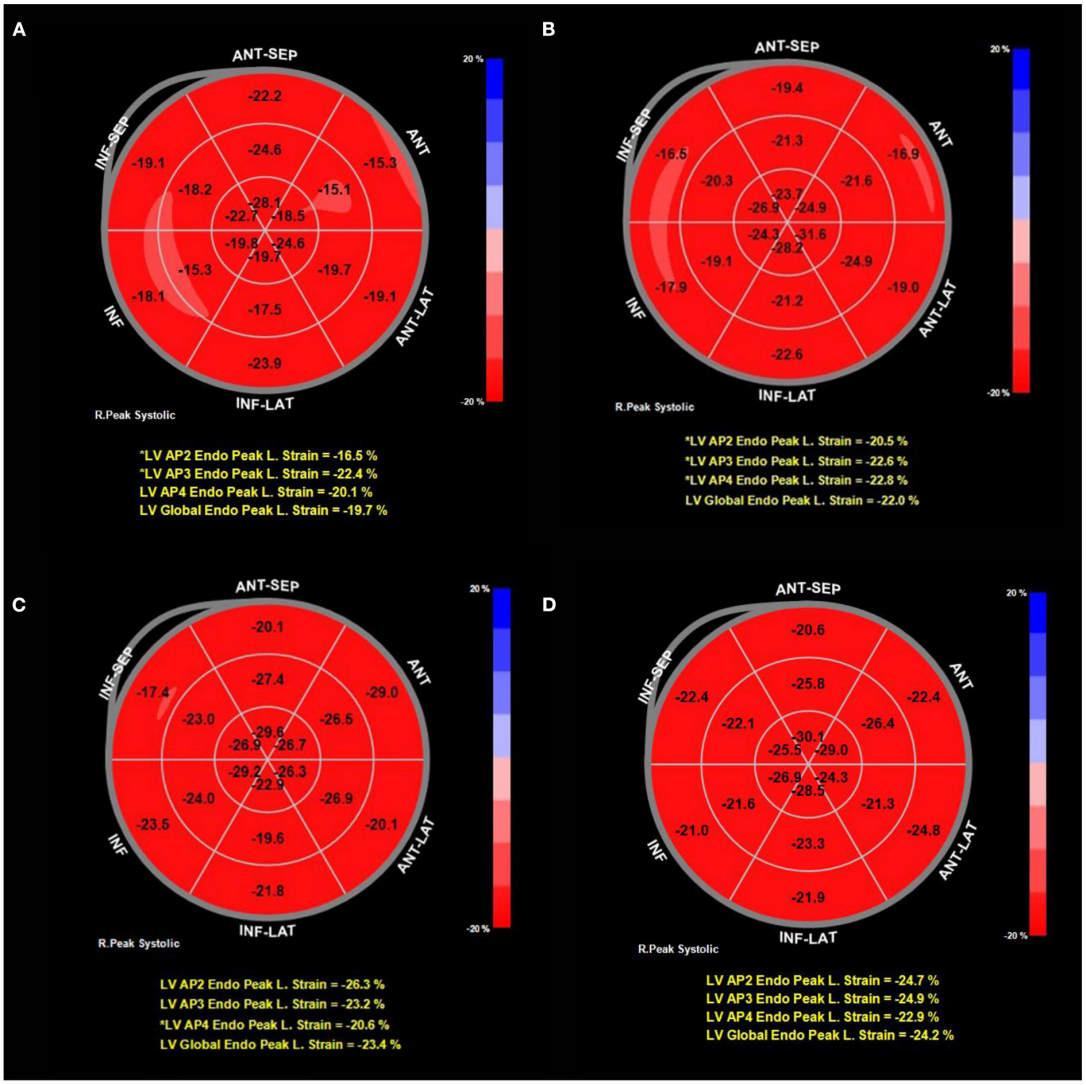
Typical bull's eye pattern of a representative single patient in the acute, convalescent and chronic stage compared to the pattern in a control patient. The longitudinal strain of subendocardium recovered gradually with the prolongation of the course of KD. **(A)** GLS-Endo in acute stage was −19.7%. **(B)** GLS-Endo in convalescent stage was −22.0%. **(C)** GLS-Endo in chronic stage was −23.4%. **(D)** GLS-Endo in the control group was −24.2%.

**Figure 2:**
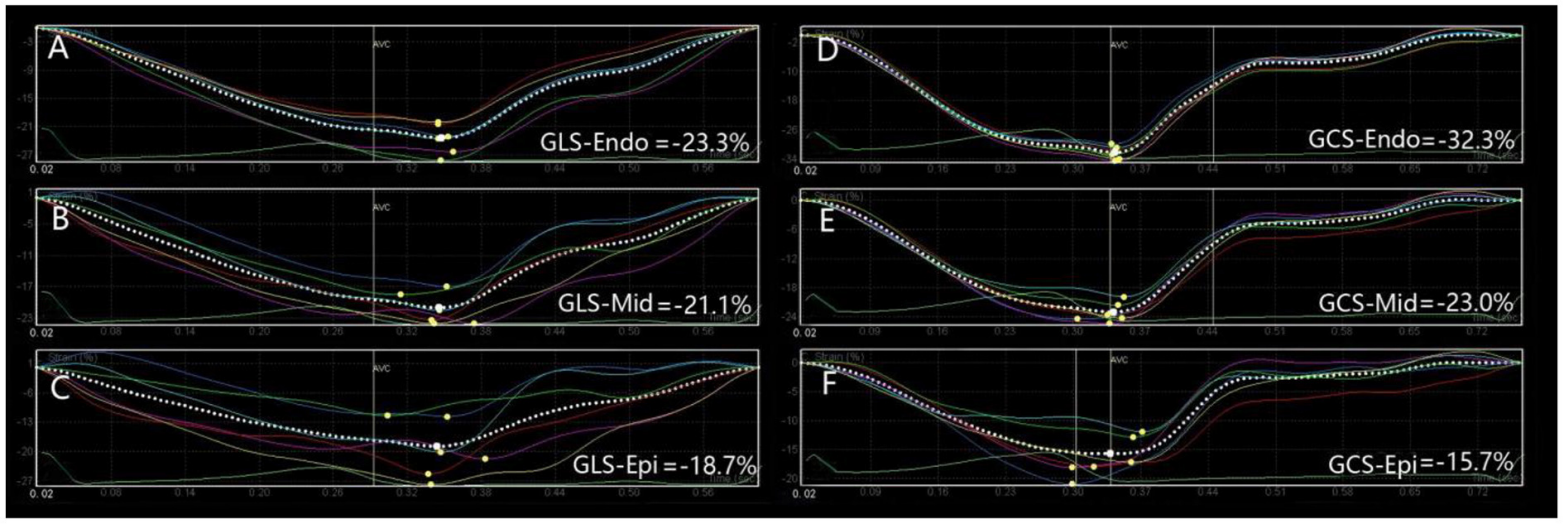
Longitudinal and circumferential strain values and strain curves of left ventricular myocardium in one representative children with chronic KD. The longitudinal and circumferential strain curves of left ventricular myocardium were basically the same. **(A)** GLS-Endo: global longitudinal strain in subendocardial myocardium; **(B)** GLS-Mid, global longitudinal in middle myocardium; **(C)** GLS-Epi, global longitudinal in subepicardial myocardium; **(D)** GCS-Endo, global circumferential strain in subendocardial myocardium; **(E)** GCS-Mid, global circumferential in middle myocardium; **(F)** GCS-Epi, global circumferential in subepicardial myocardium.

The longitudinal and circumferential strain peaks of the three layers of the left ventricular myocardium (subendocardial, middle, and subepicardial myocardium) in the KD and control groups maintained the characteristics of transmural gradient decreasing from inside to outside. Compared with the control group, LVGLS, GLS-Endo, GLS-Mid, GLS-Epi, and ΔGLS were decreased in children with KD in the acute stage; and LVGLS, GLS-Endo, GLS-Mid, GLS-Epi, ΔGLS, LVGCS, GCS-Mid, and GCS-Epi decreased in children with KD in the convalescent stage. In children with chronic KD, only GLS-Endo decreased compared with that in the control group; all the differences were statistically significant (*P* < 0.05). LVGLS, GLS-Endo, GLS-Mid, and GLS-Epi began to increase in children with convalescent KD compared with children with acute KD. They further increased in children with chronic KD (all *P* < 0.05). LVGLS, GLS-Endo, GLS-Mid, GLS-Epi, and GCS-Mid were higher in children with chronic KD than in children with convalescent KD (*P* < 0.05) (Table 3; Figures 1, 2).

**Table 3:** Comparison of 2D-STI parameters between the two groups ($\bar{x}$ ± s).

**Group**	**Cases**	**LVGLS(%)**	**GLS–Endo (%)**	**GLS–Mid (%)**	**GLS–Epi (%)**	Δ**GLS(%)**
KD group	73					
Acute stage	24	−18.56 ± 1.05^a^	−20.91 ± 1.61^a^	−18.5 ± 1.07^a^	−16.27 ± 1.17^a^	−4.63 ± 1.48^e^
Convalescent stage	26	−19.70 ± 0.69^b^	−21.98 ± 1.17^b^	−19.75 ± 0.65^b^	−17.37 ± 0.89^b^	−4.61 ± 1.23^d^
Chronic stage	23	−20.83 ± 0.79	−23.65 ± 0.83^c^	−20.43 ± 1.04	−18.41 ± 0.80	−5.23 ± 0.73
Control group	64	−21.20 ± 0.91	−24.28 ± 1.13	−20.61 ± 1.28	−18.71 ± 1.11	−5.56 ± 1.46
*F*		59.91	61.16	22.44	36.82	4.73
*P*		<0.001*	<0.001*	<0.001*	<0.001*	0.004*
**Group**	**Cases**	**LVGCS (%)**	**GCS–Endo (%)**	**GCS–Mid (%)**	**GCS–Epi (%)**	**ΔGCS(%)**
KD group	73					
Acute stage	24	−22.50 ± 2.06	−30.58 ± 3.92	−21.34 ± 2.12	−15.58 ± 1.06	−15.00 ± 3.80
Convalescent stage	26	−22.25 ± 1.43^d^	−30.32 ± 2.71	−20.88 ± 1.74^b^	−15.54 ± 1.28^d^	−14.77 ± 3.34
Chronic stage	23	−23.17 ± 2.90	−31.41 ± 5.02	−22.53 ± 2.30	−15.56 ± 2.80	−15.85 ± 4.89
Control group	64	−23.59 ± 3.08	−31.51 ± 4.57	−22.63 ± 3.38	−16.63 ± 2.73	−14.89 ± 4.54
*F*		2.05	0.65	3.25	2.45	0.34
*P*		0.110	0.583	0.024*	0.066	0.796

*P < 0.05. a, Compared with the control group, children with KD in convalescent stage and chronic stage, the differences were statistically significant (P < 0.05); b, Compared with the control group and children with KD in chronic stage, the differences were statistically significant (P < 0.05); c, d and e, Compared with the control group, the difference was statistically significant (P < 0.05).

### ROC curve of strain parameters predicting left ventricular systolic function in children with acute KD

**Figure 3:**
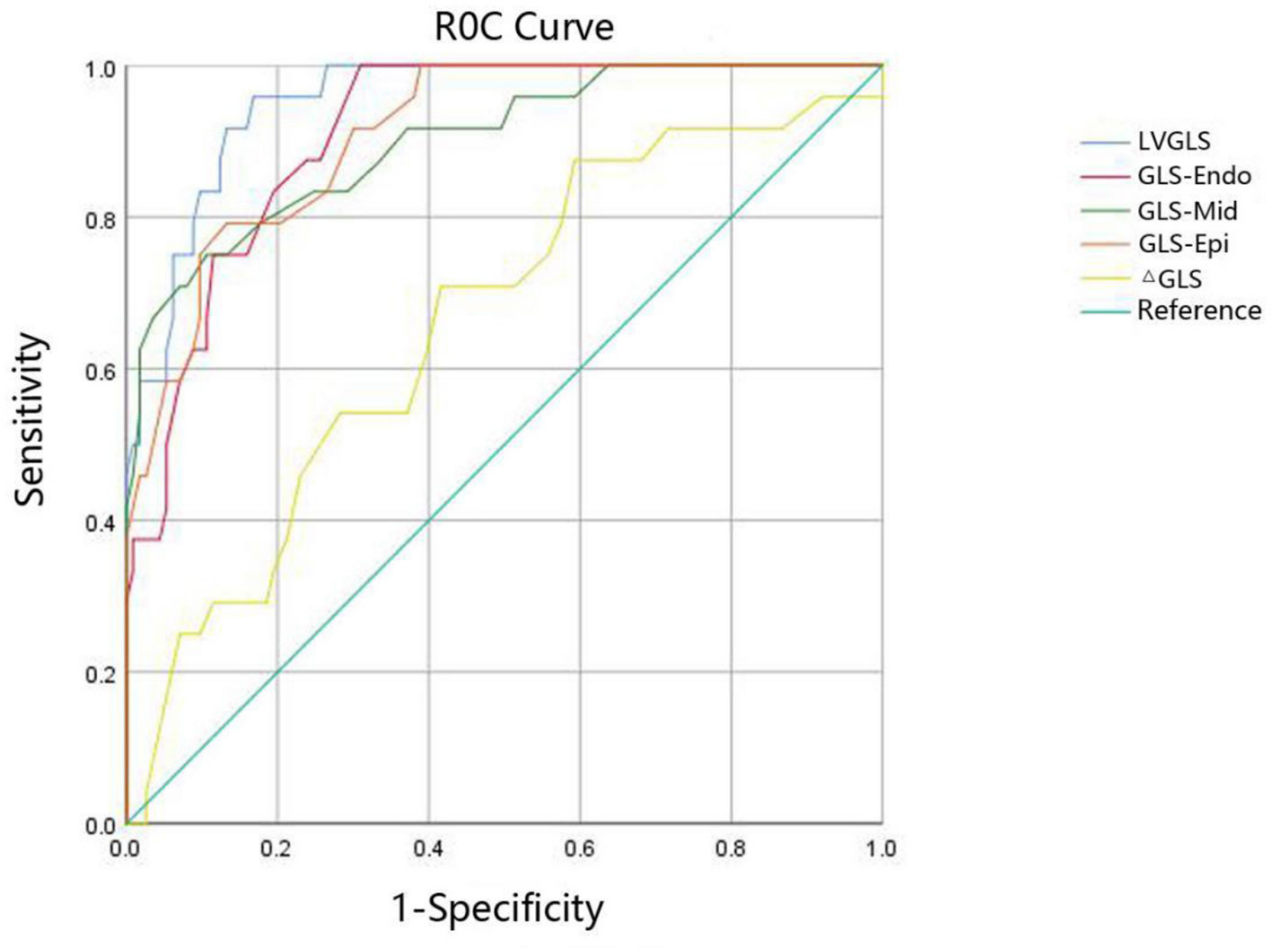
The ROC curve of strain parameters to predict the left ventricular systolic function in children with acute KD. LVGLS, left ventricular global longitudinal strain; GLS-Endo, longitudinal strain of subendocardium; GLS-Mid, longitudinal strain of middle myocardium; GLS-Epi, longitudinal strain of subepicardial myocardium; ΔGLS, longitudinal transmural strain gradient.

The ROC curve was used to calculate the AUC of left ventricular systolic function in children with acute KD. The results showed that the decrease in the longitudinal strain peak of the left ventricular three-layer myocardium, LVGLS, and ΔGLS could effectively identify the abnormal left ventricular systolic function (*P* < 0.05); the AUC of the LVGLS curve was the largest (AUC = 0.953, *P* < 0.001). When the cutoff value of LVGLS was −19.89%, the sensitivity and specificity were 95.8% and 83.2%, respectively (Figure 3; Table 4).

**Table 4:** Prediction of left ventricular systolic function by strain parameters in children with acute KD.

**Variables**	**Cut–off**	**AUC (95%CI)**	***P***	**Sensitivity**	**Specificity**
LVGLS	−19.89%	0.953 (0.919–0.987)	<0.001	95.8%	83.2%
GLS–Endo	−23.05%	0.912 (0.861–0.962)	<0.001	100.0%	69.0%
GLS–Mid	−19.15%	0.900 (0.828–0.972)	<0.001	75.0%	89.4%
GLS–Epi	−17.15%	0.910 (0.854–0.966)	<0.001	79.2%	86.7%
ΔGLS	−4.90%	0.659 (0.540–0.779)	0.014	70.8%	58.4%

## Discussion

The normal left ventricular wall comprises endocardium, myocardium, and epicardium. Also, the myocardium is divided into three layers: oblique subepicardial muscle fibers in a left-handed spiral, longitudinal subendocardium muscle fibers in a right-handed spiral, and circular middle muscle fibers (12). The longitudinal muscle fibers in the subendocardium are responsible for the longitudinal strain of the left ventricle, while those in the middle and subepicardial myocardium are responsible for circumferential and torsional movements. The strain values of the subendocardial myocardium, middle myocardium, and subepicardial myocardium obtained by the 2D-STI layered strain technique are consistent with the anatomy of the three-layer muscle band of the left ventricular myocardium, which can more accurately reflect the situation of myocardial movement (13). The structure of these myocardial fibers is unique, and each layer of the myocardium plays a different role in ventricular function. Therefore, various pathological factors may have different effects on the function of each layer of the myocardium, especially in different stages of disease development. Many previous studies (14–16) have confirmed the prevalence of abnormal myocardial histology in children with KD in the acute stage. However, few studies have been conducted on the application of the 2D-STI layered strain technique to evaluate the changes in left ventricular systolic function in children with KD in different stages.

The results showed that LVGLS, GLS-Endo, GLS-Mid, GLS-Epi, and ΔGLS were significantly lower in children with acute KD than in the control group (*P* < 0.05). The subendocardial myocardium in the spiral arrangement has poor tolerance to hypoxia due to the characteristics of the coronary artery supplying blood from the epicardium to the endocardium. When myocardial ischemia occurs, it results in microvascular dysfunction and myocardial fibrosis, thus affecting the longitudinal movement of the heart (17). Therefore, children with early KD in the acute stage can show a reduced longitudinal strain. The circumferential strain may remain normal; that is, the abnormal longitudinal strain of the myocardium during myocardial ischemia is earlier than the circumferential strain. The results showed that besides the decrease in longitudinal strain parameters, LVGCS, GCS-Mid, and GCS-Epi in children with KD in convalescence also decreased. This might be because the circumferential strain reflected the circular movement of the myocardium, and the middle myocardium was the main influencing factor. When the middle myocardium was affected by ischemia and hypoxia, the circumferential strain of the myocardium might change, and the radius of curvature of the circumferential muscle fibers was smaller than that of the longitudinal muscle fibers, and their pressure was also lower, and therefore the circumferential strain generally occurred later in the dysfunction. LVGLS, GLS-Endo, GLS-Mid, and GLS-Epi began to increase in children with convalescent KD compared with children with acute KD. They further increased in children with chronic KD (all *P* < 0.05). LVGLS, GLS-Endo, GLS-Mid, GLS-Epi, and GCS-Mid were higher in children with chronic KD than in children with convalescent KD. However, in the chronic stage, only GLS-Endo was still lower than that in the control group, suggesting that the reduction of left ventricular systolic function in children with KD was reversible, and only subendocardial myocardial ischemia was present in the chronic stage.

No specific pathological diagnosis of KD is currently available. The diagnosis still depends on clinical criteria, and it is difficult to identify some clinical symptoms in the early stage of the disease. Although some children have significant symptoms and signs of the cardiovascular system in the acute stage (11), a large number of children in the acute stage miss the optimal treatment time in the process of waiting for a diagnosis. Therefore, seeking sensitive indicators for the early detection of cardiac dysfunction in children with KD is particularly important. In this study, the ROC curve was used to calculate the stratified strain parameters so as to predict the left ventricular systolic function in children with acute KD. The results showed that the decrease in the longitudinal strain peak of the left ventricular three-layer myocardium, LVGLS, and ΔGLS could effectively identify the abnormal left ventricular systolic function (*P* < 0.05); the area under the LVGLS curve was the largest (AUC = 0.953, *P* < 0.001). When the cutoff value of LVGLS was −19.89%, the sensitivity and specificity were 95.8% and 83.2%, respectively, and the diagnostic accuracy was the highest.

Newburger et al. (18) found that LVEF in children with KD in the acute stage was lower than that in the control group, which might result from the reduced myocardial systolic function of the left ventricle. In this study, no significant difference in LVEF was found between children with KD and the control group, which was consistent with the results of Xu et al. (19). At this time, the left ventricular systolic functional strain decreased to varying degrees, suggesting that routine echocardiography could not detect abnormal systolic function early (20). With the prolongation of the disease course, the myocardial function gradually recovered. However, in our study, the GLS-Endo in chronic stage was still lower than in control group which was inconsistent with previous research (19), the reasons may be related to the number of patients, follow-up time, the duration of the disease and other factors. Therefore, when children with KD are suspected in clinic, the 2D-STI analysis should be performed as early as possible to find the subclinical changes in myocardial systolic function. In the case of insufficient time to evaluate the strain of each segment of left ventricle in children, LVGLS should be measured at least to find abnormal systolic function, so as to provide important reference information for early clinical diagnosis and timely treatment.

This study had several limitations. Since the current software cannot analyze the radial strain of the left ventricular three-layer myocardium, only the longitudinal strain and circumferential strain were analyzed. And the 2D-STI analysis required a high display definition of the endocardium and epicardium. The participants in this study were infants and young children. Although their chest wall was thin and had a good sound permeability, some image information, especially the apical image acquisition, might be lost due to the influence of examiners' experiences and some infants' rapid heart rates. Further, it affected the subsequent image analysis and also led to the failure of the inclusion of some cases in this study, resulting in a small sample size. The feasibility of the research results needs to be further confirmed using a larger sample size.

Finally, it is worth mentioning that under the COVID-19 epidemic, the British and American pediatric critical care community put forward the concept of multisystem inflammatory syndrome in children (MIS-C). The main clinical manifestations of the disease are similar to the symptoms of KD in infants under 4 years old, which can be fatal in severe cases. It is characterized by persistent fever and a range of symptoms including hypotension, involvement of multiple organs (e.g., heart, gastrointestinal tract, kidney, hematology, dermatology and neurology) and elevated inflammatory markers, but not all children develop respiratory symptoms.

## Conclusions

In conclusion, before the decrease in LVEF in children with KD, the left ventricular systolic function was damaged and the systolic function of the whole myocardium decreased to varying degrees. With the prolongation of the disease course, the myocardial function gradually recovered, but the subendocardial myocardium was still damaged. 2D-STI could identify the degree of involvement of left ventricular systolic function in children with KD in different stages by evaluating myocardial stratification strain. Among the related indexes of the myocardial stratified strain, LVGLS had high accuracy as an indicator of early systolic dysfunction of the left ventricular myocardium in children with acute KD. It also had good test efficiency and clinical application prospect.

## Data availability statement

The original contributions presented in the study are included in the article/supplementary materials, further inquiries can be directed to the corresponding author.

## Ethics statement

The studies involving human participants were reviewed and approved by the Ethics Committee of China Medical University. Written informed consent to participate in this study was provided by the participants' legal guardian/next of kin. Written informed consent was obtained from the minor(s)' legal guardian/next of kin for the publication of any potentially identifiable images or data included in this article.

## Author contributions

JH and QZ prepared the figure. JH wrote the manuscript. All authors reviewed the manuscript.

## Conflict of interest

The authors declare that the research was conducted in the absence of any commercial or financial relationships that could be construed as a potential conflict of interest.

## Publisher's note

All claims expressed in this article are solely those of the authors and do not necessarily represent those of their affiliated organizations, or those of the publisher, the editors and the reviewers. Any product that may be evaluated in this article, or claim that may be made by its manufacturer, is not guaranteed or endorsed by the publisher.
